# Hyperamylasemia Post Living Donor Nephrectomy Does Not Relate to Pain

**DOI:** 10.7759/cureus.8217

**Published:** 2020-05-21

**Authors:** Maria Irene Bellini, Rebekah S Wilson, Peter Veitch, Tim Brown, Aisling Courtney, Alexander P Maxwell, Vito D'Andrea, James McDaid

**Affiliations:** 1 Surgery, Belfast Health and Social Care Trust, Belfast, GBR; 2 Regional Transplant Unit, Belfast Health and Social Care Trust, Belfast, GBR; 3 Nephrology, Queen's University Belfast, Belfast, GBR; 4 Surgery, Sapienza University, Rome, ITA

**Keywords:** living donor nephrectomy, post-operative pain, pancreatitis, laparoscopy, hyperamylasemia

## Abstract

Introduction

The aetiology of pain after laparoscopic donor nephrectomy remains unclear. Given the proximity of the left kidney to the tail of the pancreas, we aimed to assess whether mobilisation and retrieval of the left kidney might inflame the pancreas, leading to pain and hyperamylasaemia in the post-operative period.

Patient and methods

In the present study, 16 consecutive live kidney donors were analysed in the same three months period. Amylase levels were measured on days 1 and 2. For each 24-hour period post-operatively analgesia consumption was recorded, as well as pain scores at rest on a visual analogue scale (VAS).

Results

Three out of 16 donors presented hyperamylasemia. A multiple regression analysis found levobupivacaine dose, propofol dose, transversus abdominis plane block and day 1 amylase did not significantly predict pain scores. Interestingly, body mass index significantly correlated with increased pain scores (p = 0.041). Also, increasing CO2 insufflation pressure and use of local anaesthetic infusion catheters predicted a decreased deep pain score (p = 0.036 and p = 0.037).

Conclusion

There was no correlation of amylase levels and pain scores. Pancreatitis is a rare complication of nephrectomy and no overt cases were seen in the case of donor nephrectomy.

## Introduction

Laparoscopic living donor nephrectomy (LDN) has eased the burden on the kidney transplant waiting list by making donation more attractive due to decreased pain, shorter hospital stay, earlier return to work and smaller scars [1,2]. Although less strenuous and more aesthetically pleasing than an open procedure, it is still a major operation with post-operative pain, physiological stress, morbidity and a 1 in 3500 risk of death [3]. Post-operative complications include: wound infections, urinary tract infections, ileus, iatrogenic bowel injury, splenic laceration and impaired renal function [4].

Post-operative pain may be due to port sites, incisions, organ nociception, diaphragmatic irritation and Foley catheter associated discomfort. Identifying methods to reduce pain is key as pain leads to increased analgesic requirements (specifically opioids), which has been shown to significantly increase hospital stay [5]. Opioids also entail side effects of nausea, vomiting, dizziness, constipation and respiratory depression.

In this study we investigated the possibility that in some cases, post-nephrectomy pain may be attributed to pancreatitis. This may not be recognised, as the symptoms might be attributed to another cause. As the kidneys, particularly the left, are in proximity to the pancreas, we hypothesised that during surgery the pancreas may be agitated, triggering pancreatitis.

We analysed visual analogue scale (VAS) pain scores, calculated Glasgow scores and measured amylase levels to detect mild pancreatitis contributing to post-operative pain that is currently unrecognised in this patient population.

We hypothesised that:

There is a positive correlation of VAS pain scores in relation to amylase levels.

There is a larger rise in amylase levels in left nephrectomies in comparison to right nephrectomies.

An elevated Glasgow Score will have a positive correlation with VAS pain scores.

## Materials and methods

We prospectively collected data from live kidney donors who were scheduled to have a nephrectomy in our centre over a three-month period. Patient information recorded included: age, gender, body mass index, previous surgery, past medical history and pre-op bloods (full blood count (FBC), urea and electrolytes (U+E), bone profile, fasting glucose, liver function tests (LFTs) and amylase. We documented the emotional relation of the recipient to whom the donor was donating (sibling, parent, cousin, spouse, etc.).

VAS pain scores were recorded, with a range of 0 to 10. Parameters recorded intraoperatively were: insufflation pressures, method e.g. laparoscopic, right or left nephrectomy, propofol dose (mg), use of pre-op transversus abdominis plane (TAP) block and local anaesthetic infiltration catheter.

Glasgow Score was calculated post-operatively. None of the patients had oxygen saturations below 96%. Analgesia consumption from intraoperatively until 24 hours post-op was recorded and patients were asked to estimate their pain scores at rest on a VAS 24 hours after the operation. This was categorised into incisional pain, shoulder tip pain and deep abdominal pain. This was repeated daily until discharge, recording analgesic consumption for every 24 hours period since the operation.

All variables were descriptively analysed. Where appropriate, t-test, ANOVA and regression models were used. Values were considered statistically significant if p < 0.05. Data was analysed with SPSS, version 23 for Windows (IBM Corp., Armonk, NY).

## Results

There were 16 live kidney donors, all performed laparoscopically. Three live donors were unrelated and 13 related, five were siblings, three were children and five were parents of the recipients. The mean donor age was 41.9 (SD 8.6). Six patients were female and 10 were male. Mean body mass index (BMI) was 27.7 (SD 4.5), range 20-35.4. Table 1 summarises demographic characteristics for the donors.

**Table 1:** Donors' demographic characteristics

Donor ID	Age	Gender	BMI (kg/m^2^)	Relation
1	49	M	26.4	Friend
2	28	M	24	Spouse
3	52	M	25	Spouse
4	45	F	22.4	Spouse
5	38	M	24	Parent
6	58	M	31.9	Child
7	53	F	35.4	Sibling
8	47	F	31.4	Sibling
9	38	M	23	Sibling
10	39	M	28	Parent
11	43	M	33	Parent
12	31	F	20	Child
13	43	F	26	Sibling
14	41	F	30	Parent
15	30	M	32	Parent
16	35	M	30.3	Child

There were six patients who had abdominal surgery prior to donation. No donor had a history of pancreatitis. One had a history of gallstones and another recently had a cholecystectomy. One patient had post-traumatic stress disorder, one had alcohol dependence and three had depression. There was no significant difference in days 1 and 2 total pain scores between those with and without depression (p = 0.668 and p = 0.061, respectively). The maximum Glasgow score was 1 and was either due to patient age or raised white cell count. A one-way ANOVA for days 1 and 2 total pain scores between groups for relationship of donor to recipient were not significant, p = 0.606 and p = 0.321, respectively.

The mean dose of levobupivacaine used on day 1 was 369 mg (SD 121 mg). Oral analgesia taken included: paracetamol, non-steroidal anti-inflammatory drugs (NSAIDs), oxycodone, hyoscine and butylbromide as an adjunct. Day 1 mean deep pain VAS score was 3.6 (SD 3.1), range 0 to 9.5, incisional pain score 3.6 (SD 2.7), range 0 to 8 and shoulder tip pain score 3.1 (SD 3.4), range 0 to 10. The mean dose of levobupivacaine used on day 2 was 125 mg (SD 111 mg). Day 2 mean deep pain VAS score was 2.1 (SD 2.2), range 0 to 6, incisional pain score 1.5 (SD 0.9) range 0 to 3 and shoulder tip pain score 0.9 (SD 1.4) range 0 to 5.

Three patients had a modestly elevated amylase level at 126, 155 and 159 U/L (normal range 28-100 U/L) on Day 1 post-operatively. Their corresponding pain scores can be seen in Table 2.

**Table 2:** Pain scores for the donors with day 1 hyperamylasaemia

	Amylase level	Deep pain	Incisional pain	Shoulder tip pain
Donor no. 2	126 U/L	4	8	1
Donor no. 10	155 U/L	8	6	0
Donor no. 15	159 U/L	0	0.5	2

A multiple regression was run to predict VAS day 1 deep pain scores from: day 1 levobupivacaine dose, propofol dose, BMI, transversus abdominis plane (TAP) block, local anaesthetic wound catheter, day 1 amylase and CO2 insufflation pressure. None of these variables significantly predicted VAS day 1 deep pain score p = 0.463, R2 = 0.518. Table 3 details values for each variable.

**Table 3:** Multiple regression analysis for day 1 deep VAS pain scores VAS: Visual analogue scale; TAP: Transversus abdominis plane.

	B	Significance
BMI	-0.311	0.219
CO_2 _insufflation pressure	-1.020	0.340
Propofol dose	-0.041	0.139
Day 1 amylase level	0.030	0.359
TAP block	-1.483	0.315
Local anaesthetic wound catheter use	-1.350	0.789
Day 1 levobupivacaine dose	0.011	0.485

A multiple regression was run to predict VAS day 2 deep pain scores from: BMI, local anaesthetic wound catheter use, day 2 amylase and CO2 insufflation pressure. These variables significantly predicted VAS day 2 deep pain score, p = 0.046, R2 = 0.710. Table 4 details values for each variable.

**Table 4:** Multiple regression analysis for day 2 deep VAS pain scores VAS: Visual analogue scale

	B	Significance
BMI	0.689	0.011
CO_2 _insufflation pressure	-1.533	0.014
Day 2 amylase level	-0.031	0.705
Local anaesthetic wound catheter use	-9.782	0.007

There is no positive correlation of VAS pain scores in relation to amylase levels on days 1 or 2. There is positive correlation of VAS pain scores in relation to insufflation pressures, for every 1 mmHg increase in CO2 pressure there is a decrease in day 2 VAS deep pain scores by -1.533. There was no significant correlation when analysing shoulder tip pain on days 1 and 2 (p = 0.247 and p = 0.326). The median CO2 insufflation pressure was 12 mmHg (range 10-15 mmHg). Increasing BMI predicted an increased day 2 pain score, p = 0.011. All patients had local anaesthetic wound catheter use, however it did not function in one patient. Local anaesthetic wound catheter use predicted a decreased deep pain score (p = 0.007). Eight patients had a TAP block; two were bilateral and six were unilateral on the side of the nephrectomy.

One patient had a right nephrectomy and 15 had left nephrectomies. An independent t-test found no significant difference in day 1 and day 2 amylase levels between right versus left nephrectomy, p = 0.722 and p = 0.904, respectively.

## Discussion

The primary aim of the present study was to investigate whether pain scores positively correlate with amylase levels. There were no overt cases of pancreatitis, but we detected hyperamylasaemia in three patients post-operatively. More in details, in Table 1, the donor with the highest amylase level had low pain scores in each category whilst the others had moderate to high scores. This was reinforced by multiple regression analysis, as amylase levels did not predict deep pain scores (p = 0.359 day 1 and p = 0.705 day 2). There was no significant difference in day 1 or day 2 amylase levels between right versus left nephrectomy, p = 0.722 and p = 0.904, respectively.

Day 1 pain scores for deep, incisional and shoulder tip were on average 3.6, 3.6 and 3.1, respectively. Day 2 pain scores for deep, incisional and shoulder tip were on average 2.1, 1.5 and 0.9, respectively. This reflects good pain control in the donors overall, however the range of scores on day 1 still reaches 9.5, 8 and 10, respectively. Many patients described feeling bloated, which was not eased by several modes of analgesia and only slightly dulled by buscopan. For every unit increase in BMI the regression analysis predicts an increased day 2 VAS pain score (p = 0.011), possibly due to increasing technical difficulty of the operation, requiring greater mobilisation of the viscera.

Multiple regression analysis predicted that for every 1 mmHg increase in CO2 pressure there is a decrease in day 2 VAS deep pain scores (p = 0.014). This was not significant on day 1 or when analysing shoulder tip pain on days 1 and 2 (p = 0.247 and p = 0.326). This was unexpected, as it has been previously reported that lower pneumoperitoneum pressures reduce post-operative pain. Warle et al. compared 7 mmHg vs 14 mmHg and found lower pressure resulted in a lower overall pain score, referred pain score and deep pain score [6]. There was no difference in complication rate, but it resulted in longer skin-to-skin time and potentially a poor view of the operative field due to low pressure. Our findings should be interpreted with caution because of low patient numbers. It may be that a higher-pressure pneumoperitoneum allows ideal visualisation of the operative field and possibly a more efficient nephrectomy with less movement of neighbouring viscera.

Our centre routinely conducts a TAP block prior to nephrectomy [7]. We did not analyse this further due to the time when the first pain scores were collected being 24 hours after surgery by which time the TAP block would have worn off.

A study by Azawi et al. compared TAP block versus local ropivacaine injection and found in reference to the visual analogue scale (VAS) that TAP block did not reduce pain or opioid consumption (VAS 3.05 vs 1.44 P = 0.001) [8]. They hypothesised that this difference in VAS scores was possibly due to the TAP block wearing off by the time the patient was awake. In comparison, Parikh et al. found reduced tramadol consumption and reduced VAS score up to 12 hours post op and Hosgood et al. found reduced early morphine consumption [9]. Before closure of incisions, local anaesthetic wound catheters are inserted, and injected with chirocaine or levobupivacaine. When back on the ward these are attached to a bag of 0.125% levobupivacaine, which commonly infuses at a rate of 10 ml/hour for 24-48 hours after nephrectomy. Use of these catheters predicted a decreased deep pain score (p = 0.007) on day 2 which indicates their effectiveness and allows continuous analgesia to the TAP.

A literature review discovered a total of 16 cases of pancreatitis post-nephrectomy for urologic indication, though none for transplant donor nephrectomy. More details are shown in Table 5 [1, 4, 8-12].

**Table 5:** Number of cases of pancreatitis documented as a post-operative event during nephrectomy

Authors	Number of pancreatitis events	Nephrectomy procedure
Al-Ali et al. [13]	1	Open
Dunn et al. [10]	1	Open
Hawasli et al. [11]	1	Laparoscopic
Jacobs et al. [5]	4	Laparoscopic
Kortram et al. [1]	3	Minimally invasive
Pietrow et al. [4]	2	Hand assisted
Varkarakis et al. [12]	4	Adrenalectomy and laparoscopic nephrectomy

We felt that given the longer length of vessels needed for donor nephrectomy versus urologic nephrectomy, there might be more pancreatic mobilisation and pancreatitis in the donor operation. Incidence of pancreatitis in urologic series varied from 0.3% to 3.3%. Varkarakis et al. documented four pancreatic injuries during urological surgery: two during adrenalectomy and two as a result of nephrectomy [12]. Two of these were associated with epigastric pain and hyperamylasaemia and the overall incidence was 0.44%. Al-Ali et al. presented a case of acute pancreatitis after a right radical transperitoneal open nephrectomy [13]. The patient developed abdominal and back pain, jaundice and fever and was admitted to ITU for 15 days.

Other case reports including Devlin et al. and Gottschling et al. have presented cases of propofol-induced pancreatitis, unrelated to surgery [14,15]. In Gottschling’s case report, a young girl was described who was given propofol for an MRI scan and then developed pancreatitis within hours. A large majority of patients having a nephrectomy are anaesthetised with propofol, so differentiating between propofol and abdominal surgery, as the cause of pancreatitis, may be challenging.

Wang et al. compared VAS in living donors and patients undergoing similar surgical procedures for removal of renal cell carcinoma [16]. They found that in donors, time to tramadol request was quicker, amount of tramadol consumed was higher and VAS scores were higher in the first 8 hours. There was no clear reason for this, but they proposed it, may be due to difference in the perception of pain, as the donor is only benefiting someone else whereas cancer patients benefit directly. They speculated that the donor may experience more pain as it is contributed to psychologically. They also felt that doctors and nurses might pay more attention to donors, resulting in earlier administration and more analgesia. We investigated whether there was a significant difference between the type of relation of the donor to the recipient and pain scores, hypothesising that those who were more closely related to the recipient might have lower pain scores than those who were more distantly related, however, we found no significant difference.

## Conclusions

In conclusion, in laparoscopic LDN post-operative pain remains of unknown aetiology. We investigated the possibility that post-nephrectomy pain could be attributed to hyperamylasemia as the kidneys, particularly the left, are in proximity to the pancreas, but this is not the case as post-operative pain scores are not correlated with amylase levels, while increasing BMI and reduced insufflation pressure correlated with increased deep pain scores. Local anaesthetic wound catheter use significantly reduced deep pain scores.
